# Conservative Treatment of a Scald in a Puppy

**Published:** 2009-10-05

**Authors:** Elina Dahlqvist, Andrew Lindford, Virve Koljonen

**Affiliations:** ^a^Korson Eläinlääkäriasema KIRSU, Urpiaisentie 1B, 01450 Vantaa, Finland; ^b^Department of Plastic Surgery, Helsinki University Hospital, Helsinki, Finland

## Abstract

**Objective:** We describe a case of a large scald in a 9-month-old puppy that was treated conservatively. **Case:** On admission to the private veterinary clinic, the scald was already 2 weeks postinjury, with no significant signs of healing. The burn surgeon from the University Hospital was consulted regarding the appropriate treatment protocol. Advice was given recommending careful showering of the scald wound, removal of debris, and loose hair, as well as the treatment of infection. This resulted in successful healing of the burn wound within a week. **Discussion:** This case highlights that correct treatment of a burn injury from the outset and successful cooperation between medical and veterinary colleagues can achieve the best possible result in human and in canine burn patients.

## CASE

A 9-month-old Welsh springer spaniel, weighing 16.5 kg, was referred by its owners to a private veterinary clinic after having suffered a scald burn 2 weeks previously.

The scald occurred when the puppy jumped at her owner who at the time had a kettle of boiling water in her hands. Immediately after the accident, the puppy was doused with cold water and referred to an on-call veterinary clinic, where ointment was applied to the burned areas. No initial showering of the burned areas, shaving of the hairs to reveal the extent of the injury, or removal of blisters or loose hair was performed.

Two weeks later, the owners referred the puppy to the private veterinary clinic, Kirsu in Vantaa, Finland, because of the absence of healing and overall worsening of the puppy's general condition. On admission to this private veterinary clinic, the puppy was in pain, apathetic, and lacking energy.

**Figure 1:**
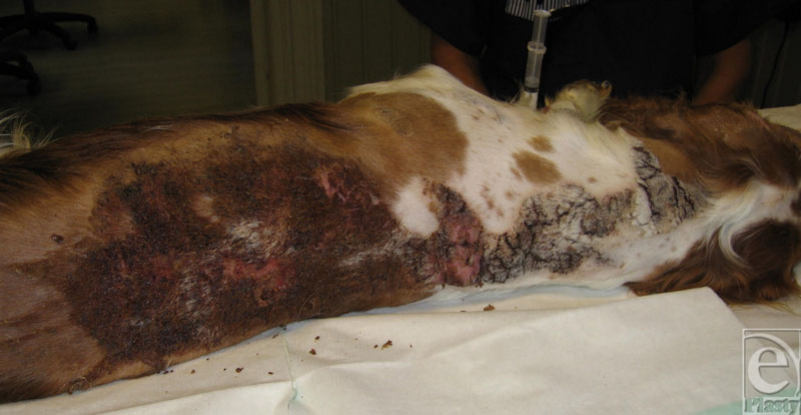
A 9-month-old Welsh springer spaniel with a 17-day-old large scald burn; burn area is 15% of the total body surface area. Note the dried debris covering the injured area.

Examination of the puppy revealed extensive burned areas affecting approximately 15% of the total body surface area (Fig 1). The injured areas were on the left side of the head around the eye and ear, the neck, left scapula, and the dorsal thoracic region. It was initially difficult to ascertain the extent of the affected areas because of the covering of fur and debris. The most severe and deepest lesions were over the back. The history of the burn injury mechanism was consistent with the resultant burn pattern observed.

Antibiotic therapy, amoxicillin-clavulanic acid (Synulox vet 140 mg/mL 2 mL sc; Pfizer Oy, Animal Health, Espoo, Finland) was initiated prophylactically against the potential infection. Meloxicam (Metacam 5 mg/mL 0.6 mL sc; Boehringer Ingelheim Vetmedica GmbH, Ingelheim/Rhein, Germany) was administered for pain control. In addition, 500~mL of warmed Ringer's lactate fluid was given. The puppy was sedated with medetomidine hydrochloride (Domitor 10 mg/mL 0.2 mL im; Orion Pharma, Espoo, Finland) and butorphanol (Torbugesic 10 mg/mL vet inj 0.2 cc im; Ft Dodge Animal Health, Ft Dodge, Iowa). The sedation was maintained with boluses of propofol (PropoVet 10 mg/mL, total 15 mL iv; Abbott Laboratories Ltd, Kent, England) while preserving spontaneous, breathing.

The puppy was then shaved and the burned areas were observed to be covered with thick debris, which was partly dried and partly covered by yellowish exudates and pus. The surrounding hairs were also coated with foul-smelling debris. Wound cleansing was carried out with Betadine (povidone-iodine; Oy Leiras Finland Ab, Helsinki, Finland) diluted with normal saline. As much of the debris and as many of the remaining hairs as possible were gently removed. The burn wound was then covered with 1% silver sulfadiazine (Flamazine; Smith & Nephew, Hull, England) dressings. This process lasted for 3 hours. The owners were given instructions to hydrate the areas and change the silver sulfadiazine bandages daily. Synulox 200 mg bid and tramadol hydrochloride (Tramal 100 mg/mL 7 drops bid) were prescribed. A plastic surgeon specializing in human burn care at the University Hospital was also consulted regarding the appropriate future treatment protocol.

After 4 days, the puppy was again sedated. The skin was surprisingly well healed and there was already evidence of epithelialization over the face. The animal was showered with warm water for 10 minutes under a normal shower. The surrounding areas were also showered. All of the debris and loose hairs were gently removed.

**Figure 2:**
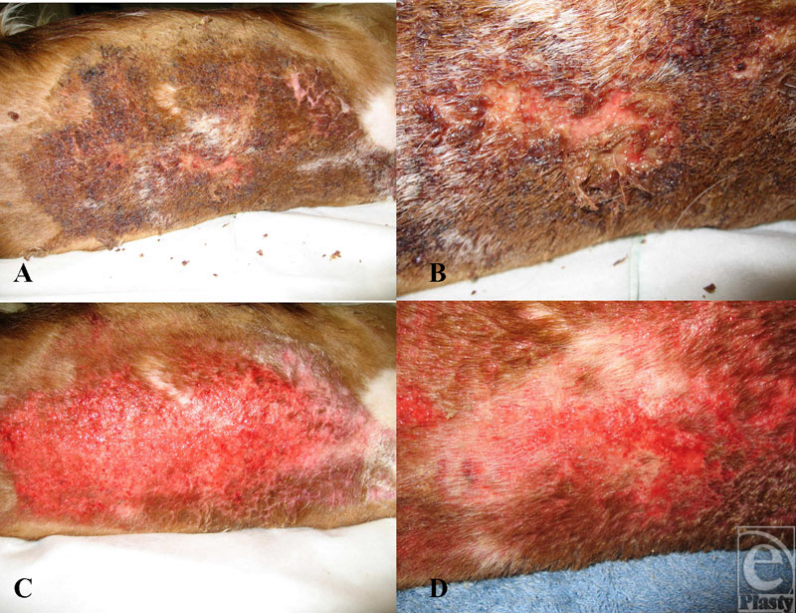
(A) Before cleansing of the wound bed, the burn area and the surrounding hair were covered with thick, foul-smelling debris, which was partly dry. (B) Close-up of the deepest region in the central area of the wound. (C) After showering and manual debridement, the wound bed looks healthy, neck shows weak reepithelialization. (D) Close-up of the deepest parts of the burn after cleansing.

The plastic surgeon estimated that the facial burns were superficial because of their epithelialization. The depth of the burn injury on the back was estimated to be of mostly superficial, second degree, with full-thickness patches in the central area of the burn. Operative treatment options were also discussed. After showering, the animal was placed onto a table and wound cleansing was continued. The remaining debris covering the wound bed was meticulously removed with tweezers. This process took altogether 1.5 hours (Fig 2). The burn wound was then covered with 1% silver sulfadiazine dressings. Removal of the thick, dry debris in the neck area revealed epithelialization. The owners were then given instructions to continue daily showering in order to keep the wound bed clean. The first photographs were taken during this appointment.

**Figure 3:**
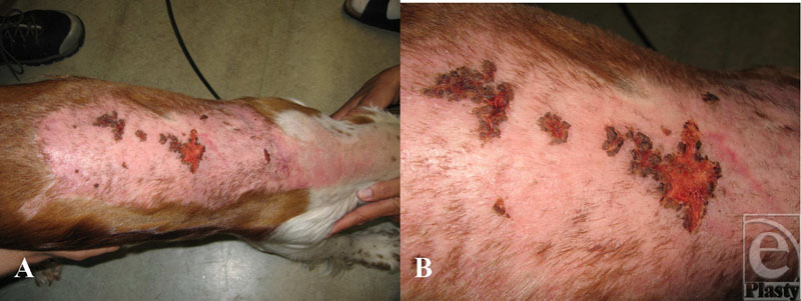
One week after thorough cleansing and debridement. (A) Almost the whole burn wound has reepithelialized; new regrown hairs are noted over the back. (B) Close-up of the deepest parts of the burn, with some still nonhealed patches.

At a planned outpatient clinic visit a week later, the puppy was playful and energetic. Some of the burned areas were now covered with regrown hairs. Only very small areas over the centre of the burn had not yet epithelialized (Fig 3). These areas were, however, so small that a decision was made to continue conservative treatment. The owners were given directions to regularly apply moisturizing cream at least twice a day. They were also advised that the deeper burn areas would need moisturizing more frequently and for a longer period of time. They were also further warned that the healed skin is more vulnerable to sunburns than regular skin and maybe more allergic to sunscreens and other skin products.

**Figure 4:**
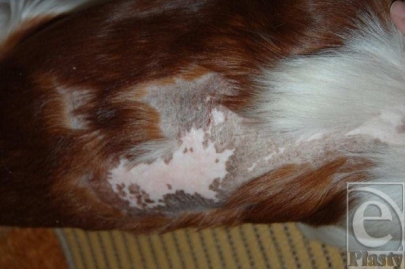
Nine months postinjury, the skin coverage is intact and strong. The central area of the burn injury is hairless, indicating deep cell destruction of the hair follicles. The most severely burned areas are vitiliginous because of damage of the melanin-producing cells.

A control photograph taken by the owners 9 months later (Fig 4) showed that parts of the burned area had regrown fur, the deepest wound area was hairless, and depigmentation could be seen in the burned parts of the body. Otherwise the puppy was healthy.

## DISCUSSION

Readers of this journal will be familiar with previous articles that have featured animals, especially canines. Some of the most profound advances in the understanding of burn injury pathophysiology have resulted from experimental canine studies.^1^,^2^ The Parkland formula, described by Baxter and Shires, was deducted from canine experiments.^1^

It may seem strange that a human burn surgeon became involved in such a case. In fact, the university veterinary clinic was first consulted, but it simply advocated the involvement of a human burn surgeon. From a burn surgeon's point of view, this was an interesting case in several ways because it provided, for example, an opportunity to treat a patient of another species and also as a tribute to the previously mentioned canine contribution to modern burn care development.

In this current report, a puppy dog was referred by its owners to a private veterinary clinic 2 weeks after the initial injury because of delayed healing. The reason for poor healing was partly due to the incomplete management of the burned areas. There appear to be no other articles in the literature concerning the conservative treatment of scalds in puppy dogs. In fact, thermal wounds are relatively uncommon in veterinary practice and most of them are the result of accidental burns occurring at veterinary practices. These cases are associated with the application of supplemental heat to treat or prevent hypothermia in patients under or recovering from general anesthesia.^3^ This of course does not mean that house pets do not encounter burn injuries. Young puppies in particular may be at greater risk for burn injuries because of their natural curiosity, impulsiveness, and less acute perception of dangerous situations, as are their human counterparts, especially male children.^4^

Thermal energy (ie, heat) is transferred from high-energy molecules to molecules of lower energy via conduction in the tissues. When considering the effect of heat to a single cell, 2 factors contribute to the degree of tissue damage—the temperature to which the cell is exposed and the length of time the heat is sustained. These 2 factors collectively determine the degree of cell damage.^5^ If the cell is rapidly cooled, most of the heat-induced damages are avoided,^6^ which is the rationale behind the suggestion of cooling the burned areas with hand-warm water immediately after the injury.

Burns are classified as first degree; superficial and deep second degree; and third degree, depending upon the depth of thermal injury. In full-thickness burns in animals, hair may be plucked out easily with tissue forceps.^7^ The latter 2 do not heal without surgical intervention. Even in the same burn wound area, there are typically different degrees of burn injuries.^8^ The estimation of the burn wound (ie, depth), which is essential in the management of burns, has traditionally been conducted by clinical evaluation, which has problems of subjectivity.^9^ Accurate burn depth assessment is important for determining the appropriate treatment plan for burn victims.

The burn surface area is estimated by the “rule of 9s,” but adapting this rule to dogs is not accurate, because when considering the elastic redundant skin covering many dogs, there is a tendency to overestimate the surface area involved.^3^ As a general rule, fluid therapy is considered for the burn patient with more than 20% of the body surface area involved.^3^ Burn formulas designed for severely burned human patients have been adapted for veterinary use. Monitoring the body weight, central venous pressure, and urine output, as in humans, assists in regulating fluid therapy.^10^

All burns are painful. The pain sensation is mediated through inflammatory and chemical mediators,^11^,^12^ as well as uncovering of the nerve endings. In human studies, 2 kinds of pain have been established. The continuous “nagging” background pain, and the other, intense, unbearable pain and anxiety associated with dressing changes.^13^,^14^ Many veterinarians would prefer general anesthesia as opposed to true sedation during the emergency assessment.^15^ This is because most animals that are even truly sedated are still arousable. General anesthesia is also preferred to avoid excessive pain, which has been detrimental to animals leading to the windup phenomenon, adverse sympathetic stimulation, and immunosuppression.^16^ For patients with possible cooperation problems, such as the puppy described in this case report, dressing changes under general anesthesia are preferred, at least during the initial few visits, to control the pain associated with dressing changes and to allow thorough cleansing of the wound. This was also of further importance in this case because the attending burn surgeon (V.K.) suffered from mild cynophobia.

The puppy's healing was complicated because of fundamental flaws in the initial management. The extent of the injury was not properly revealed at the time of presentation; if done so, the initial treatment would have been different. In addition, inadequate cleansing of the wound resulted in the accumulation of bacteria and even pus formation. The large burn area, remaining debris, and accumulated bacteria caused the puppy to become systemically unwell, necessitating treatment with antibiotics. The thorough cleansing of the burn surface further helped diminish the bacterial load and prepare the wound bed for rapid epithelialization. Topical silver sulfadiazine seems to be the treatment of choice for burn wounds in larger and smaller animals.^3^,^17^

This case report highlights that even relatively deep burns have a tendency to heal well because of the excellent blood supply and high density of epithelial appendages in the skin of a dog. The burn injury in this case eventually healed rapidly once the wound bed was cleansed of foreign material (dead loose hair, organic and inorganic debris), burn eschar, and previously applied ointments and following the concomitant treatment of infection with systemic and local antibiotics.

In conclusion, the cooperation of a veterinary surgeon and a human burn surgeon combined the best knowledge of 2 different professions to enable the swift and satisfactory healing of a burn injury in a puppy.

## References

[1] Baxter CR, Shires T (1968). Physiological response to crystalloid resuscitation of severe burns. Ann N Y Acad Sci.

[2] Harrison WG, Blalock A (1932). A study of the cause of death following burns. Ann Surg.

[3] Pavletic MM, Trout NJ (2006). Bullet, bite, and burn wounds in dogs and cats. Vet Clin North Am.

[4] Papp A, Rytkonen T, Koljonen V, Vuola J (2008). Paediatric ICU burns in Finland, 1994–2004. Burns.

[5] Moritz A, Henriques R (1947). Studies on thermal injury, part II. Am J Pathol.

[6] Raine TJ, Heggers JP, Robson MC, London MD, Johns L (1981). Cooling the burn wound to maintain microcirculation. J Trauma.

[7] Pavletic MM (1999). Management of Specific Wounds. Atlas of Small Animal Reconstructive Surgery.

[8] Jackson DM (1953). [The diagnosis of the depth of burning]. Br J Surg.

[9] Heimbach D, Engrav L, Grube B, Marvin J (1992). Burn depth: a review. World J Surg.

[10] Dhupa N, Wingfield W, Raffe M (2002). Burn injury. The Veterinary ICU Book.

[11] Summer GJ, Romero-Sandoval EA, Bogen O, Dina OA, Khasar SG, Levine JD (2008). Proinflammatory cytokines mediating burn-injury pain. Pain.

[12] Sasaki M, Obata H, Kawahara K, Saito S, Goto F (2006). Peripheral 5-HT2A receptor antagonism attenuates primary thermal hyperalgesia and secondary mechanical allodynia after thermal injury in rats. Pain.

[13] Frenay MC, Faymonville ME, Devlieger S, Albert A, Vanderkelen A (2001). Psychological approaches during dressing changes of burned patients: a prospective randomised study comparing hypnosis against stress reducing strategy. Burns.

[14] Aaron LA, Patterson DR, Finch CP, Carrougher GJ, Heimbach DM (2001). The utility of a burn specific measure of pain anxiety to prospectively predict pain and function: a comparative analysis. Burns.

[15] Campbell VL (2005). Anesthetic protocols for common emergencies. Vet Clin North Am.

[16] Muir W, Gaynor J (2002). Handbook of Veterinary Pain Management.

[17] Hanson RR (2005). Management of burn injuries in the horse. Vet Clin North Am Equine Pract.

